# Author Correction: Parenteral BCG vaccine induces lung-resident memory macrophages and trained immunity via the gut–lung axis

**DOI:** 10.1038/s41590-023-01525-x

**Published:** 2023-05-09

**Authors:** Mangalakumari Jeyanathan, Maryam Vaseghi-Shanjani, Sam Afkhami, Jensine A. Grondin, Alisha Kang, Michael R. D’Agostino, Yushi Yao, Shreya Jain, Anna Zganiacz, Zachary Kroezen, Meera Shanmuganathan, Ramandeep Singh, Anna Dvorkin-Gheva, Philip Britz-McKibbin, Waliul I. Khan, Zhou Xing

**Affiliations:** 1 McMaster Immunology Research Centre, M. G. DeGroote Institute for Infectious Disease Research and Department of Medicine, McMaster University, Hamilton, Ontario Canada; 2 Farncombe Family Digestive Health Research Institute and Department of Pathology and Molecular Medicine, McMaster University, Hamilton, Ontario Canada; 3 Department of Immunology, Zhejiang University, Zhejiang, China; 4 Department of Chemistry and Chemical Biology, McMaster University, Hamilton, Ontario Canada

**Keywords:** Tuberculosis, Live attenuated vaccines, Alveolar macrophages

Correction to: *Nature Immunology* 10.1038/s41590-022-01354-4. Published online 1 December 2022.

In the version of this article originally published, the Figure 3g dot plot panel for PBS group 14 dpi was incorrect. However, the numerical data representing the frequencies identified in this dot plot remain correct. We have now replaced this dot plot with the correct one (original and revised panels are shown as Figs. 1 and 2 below). This correction does not affect either the total number of CD4^+^Ag85B^+^ (airway) cells reported in Figure 3i or the conclusion of the article.

**Fig. 1:**
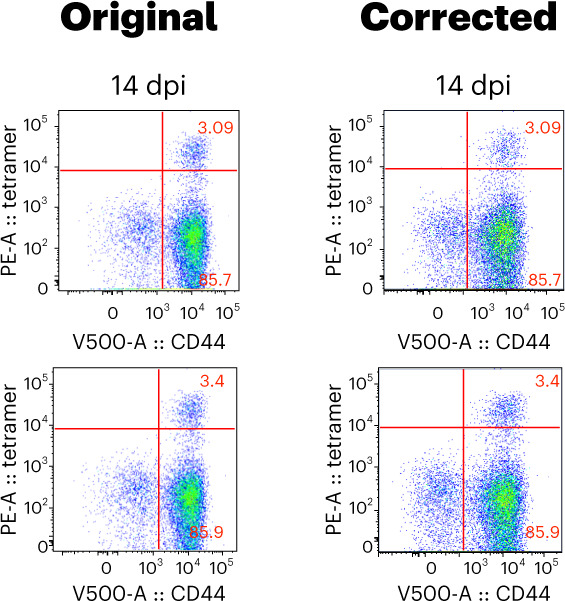
Original and corrected Fig. 3g.

