# Supplementary material for: Parenteral BCG vaccine induces lung-resident memory macrophages and trained immunity via the gut–lung axis
Source: Nat Immunol. 2022 Dec 1;23(12):1687–702. doi: 10.1038/s41590-022-01354-4 (PMC9747617; doi:10.1038/s41590-022-01354-4)
Supplement: Supplementary file 1 — Reporting Summary [file 41590_2022_1354_MOESM1_ESM.pdf]

Corresponding author(s): Zhou Xing

Last updated by author(s): Sept 19, 2022

Reporting Summary

Nature Portfolio wishes to improve the reproducibility of the work that we publish. This form provides structure for consistency and transparency in reporting. For further information on Nature Portfolio policies, see our Editorial Policies and the Editorial Policy Checklist.

Statistics

For all statistical analyses, confirm that the following items are present in the figure legend, table legend, main text, or Methods section.

n/a	Confirmed	
☐	☒	The exact sample size (*n*) for each experimental group/condition, given as a discrete number and unit of measurement
☐	☒	A statement on whether measurements were taken from distinct samples or whether the same sample was measured repeatedly
☐	☒	The statistical test(s) used AND whether they are one- or two-sided<br>*Only common tests should be described solely by name; describe more complex techniques in the Methods section.*
☐	☒	A description of all covariates tested
☐	☒	A description of any assumptions or corrections, such as tests of normality and adjustment for multiple comparisons
☐	☒	A full description of the statistical parameters including central tendency (e.g. means) or other basic estimates (e.g. regression coefficient) AND variation (e.g. standard deviation) or associated estimates of uncertainty (e.g. confidence intervals)
☐	☒	For null hypothesis testing, the test statistic (e.g. *F*, *t*, *r*) with confidence intervals, effect sizes, degrees of freedom and *P* value noted<br>*Give P values as exact values whenever suitable.*
☒	☐	For Bayesian analysis, information on the choice of priors and Markov chain Monte Carlo settings
☒	☐	For hierarchical and complex designs, identification of the appropriate level for tests and full reporting of outcomes
☒	☐	Estimates of effect sizes (e.g. Cohen's *d*, Pearson's *r*), indicating how they were calculated

Our web collection on statistics for biologists contains articles on many of the points above.

Software and code

Policy information about availability of computer code

Data collection	Immunostained cell were collected using BD LSR II or BD LSRFortessa flow cytometer by FACSdiva (Version 8.0)<br>Luminex plates were read on a MagPix reader<br>Cell morphology was studied by bright field microscopy-EVOS cell imaging system<br>Images of representative micrographs were taken under a Zeiss Axio Imager 2 Research Microscope using Zen digital imaging software (Version 2.3)<br>Real-time cell metabolism of alveolar macrophages was determined by using the Seahorse XF<br>Metabolic profiling by capillary electrophoresis coupled to time-of-flight mass spectrometry (CE-TOF-MS).
Data analysis	Flow cytometry data were analyzed by FlowJo (Version 10.8.1).<br>Concentrations of cytokines/chemokines determined by using a xPONENT software (Version 4.2)<br>Data of metabolic assay of alveolar macrophages were analyzed using Wave Desktop software version 2.6<br>Prediction of functional profiles of the bacterial communities by PICRUSt2<br>KEGG Orthology Database (https://www.genome.jp/kegg/ko.html) was used to annotate and categorize these terms, using the 2nd and the 3rd levels of the database, including terms from BRITE hierarchy, and pathways.<br>RNAseq data were analyzed by Edge R (Version 3.34.0), limma package (Version 3.48.1), rgl package (Version 0.106.8) (https://cran.r-project.org/web/package/rgl/index.html), gplots (Version 3.1.1) (https://cran.r-project.org/web/packages/gplots/index.html).<br>Microbiome data were analyzed by DADA2 and taxonomy was assigned using the SILVA database (Version 1.3.2)<br>Statistical analysis of microbiome was performed using a web-based platform MicrobiomeAnalyst (www.microbiomeanalyst.ca)<br>Metabolite raw CE-TOF-MS data were processed using Mass-Hunter Workstation Qualitative Analysis software (version B.06.00, Agilent Technologies, 2012)

Pathway analysis (targeted) and multivariate data analysis, including partial least-squares-discriminant analysis (PLS-DA) were performed using Metaboanalyst 5.0 (www.metaboanalyst.ca).
Statistical tests were performed using Graphpad Prism (Version 9.3.1)

For manuscripts utilizing custom algorithms or software that are central to the research but not yet described in published literature, software must be made available to editors and reviewers. We strongly encourage code deposition in a community repository (e.g. GitHub). See the Nature Portfolio guidelines for submitting code & software for further information.

Data

Policy information about availability of data

All manuscripts must include a data availability statement. This statement should provide the following information, where applicable:

- Accession codes, unique identifiers, or web links for publicly available datasets
- A description of any restrictions on data availability
- For clinical datasets or third party data, please ensure that the statement adheres to our policy

Raw RNA-seq data are available in the NCBI's GEO (http://www.ncbi.nlm.nih.gove/geo/) under accession No GSE213343. Raw data files for the RNA microarray analysis of intranasally adenoviral (Ad)-vaccinated mice were previously published (Yao, Y et al. Cell175, 1634-1650.e17 (2018) and can be obtained under accession No GSE118512. Microbiome sequencing data and metabolomic data can be obtained from Mendeley Data at https://doi.org/10.17632/bvvfvz67z6. The further data supporting the main findings are provided in accompanying Extended Data figures. Additional information is available via the correspondence author upon reasonable request.

Human research participants

Policy information about studies involving human research participants and Sex and Gender in Research.

Reporting on sex and gender	*Use the terms sex (biological attribute) and gender (shaped by social and cultural circumstances) carefully in order to avoid confusing both terms. Indicate if findings apply to only one sex or gender; describe whether sex and gender were considered in study design whether sex and/or gender was determined based on self-reporting or assigned and methods used. Provide in the source data disaggregated sex and gender data where this information has been collected, and consent has been obtained for sharing of individual-level data; provide overall numbers in this Reporting Summary. Please state if this information has not been collected. Report sex- and gender-based analyses where performed, justify reasons for lack of sex- and gender-based analysis.*
Population characteristics	*Describe the covariate-relevant population characteristics of the human research participants (e.g. age, genotypic information, past and current diagnosis and treatment categories). If you filled out the behavioural & social sciences study design questions and have nothing to add here, write "See above."*
Recruitment	*Describe how participants were recruited. Outline any potential self-selection bias or other biases that may be present and how these are likely to impact results.*
Ethics oversight	*Identify the organization(s) that approved the study protocol.*

Note that full information on the approval of the study protocol must also be provided in the manuscript.

Field-specific reporting

Please select the one below that is the best fit for your research. If you are not sure, read the appropriate sections before making your selection.

☒ Life sciences ☐ Behavioural & social sciences ☐ Ecological, evolutionary & environmental sciences

For a reference copy of the document with all sections, see nature.com/documents/nr-reporting-summary-flat.pdf

Life sciences study design

All studies must disclose on these points even when the disclosure is negative.

Sample size	No statistical methods were used to pre-determine sample sizes, but our sample sizes are similar to those reported in previous publications (Moorlag, S. J. C. F. M et al. Cell Rep. 31, 1076634 (2020); Guillon, A et al. JCI insight 5, (2020); Trompette, A et al. Immunity 48, 992-1005.e8 (2018) . Data distribution was assumed to be normal, but this was not formally tested. Data collection and analysis, except histological analysis, were not performed blind to the conditions of the experiments.
Data exclusions	no data points were excluded from analysis.
Replication	Most of the key experiments were repeated at least once more to establish the reproducibility of the findings, and such details were provided in individual figure legend.

Randomization	Animals were assigned to experimental groups at random.
Blinding	Since all data were included and objective analysis was carried out, data collection and analysis were not performed by researchers blinded to the conditions of the experiments except for histological analysis of the gut sections which was scored independently by two researchers blinded to the experimental groups.

Reporting for specific materials, systems and methods

We require information from authors about some types of materials, experimental systems and methods used in many studies. Here, indicate whether each material, system or method listed is relevant to your study. If you are not sure if a list item applies to your research, read the appropriate section before selecting a response.

Materials & experimental systems

n/a	Involved in the study	
☐	☒	Antibodies
☒	☐	Eukaryotic cell lines
☒	☐	Palaeontology and archaeology
☐	☒	Animals and other organisms
☒	☐	Clinical data
☒	☐	Dual use research of concern

Methods

n/a	Involved in the study	
☒	☐	ChIP-seq
☐	☒	Flow cytometry
☒	☐	MRI-based neuroimaging

Antibodies

Antibodies used	Following antibodies were used. The details on their supplier names, catalog numbers, clone names etc are provided in Supplementary Table 5 of Methods section.<br><br>All antibodies for FACS were purchased from BD Bioscience unless otherwise specified: anti-CD45-APC-Cy7 (clone 30-FII), anti-CD11b-PE-Cy7 (clone Ml/70), anti-CDllc-APC (clone HL3), anti-MHC II-Alexa Flour 700 (clone MS/114.15.2; eBioscience), anti-CD3-V450 (clone 17A2), anti-Ly6C-Biotin (clone HKl.4; Biolegend), Streptavidin-Qdot800 (lnvitrogen), anti-CD24-BV650 (clone Ml/69), anti-CD64-PE (clone X54-5/7.I; Biolegend), anti-Ly6G-BV605 (clone 1A8), anti-Siglec-F-PE-CF594 (clone ES0-2440), anti-CD11c-BV711 (clone HL3), anti-F4/80-e-Flour450 (clone BM8), anti-CD282(TLR2)-BV421 (clone CB225), anti-TNFalpha-PerCPCyS.5 (clone MP6-XT22) and anti-lL6-PE (clone MP5-20F3), anti-CD3e (clone 145-2CII, anti-Ly6G &Ly6C (cloneRB6-8C5), anti-CDllb (Clone Ml/70), anti-CD45R/B220 (clone RA3-6B2) and anti-Ly76 (erythroid cells) (clone-mTer-119), anti-c-kit-APC-Cy7 (clone 2B8), anti-Sca-l-PE-Cy7 (clone EB-161.7), anti-CD150-e-Fluor450 (clone mShadlS0), anti-CD48-PerCP-Efluor710 (clone HM48-l), anti-CD34-FITC (clone RAM34) and anti-Flt3-PE (clone A2F10.l), anti-CD3-V450 (clone 17A2), anti-CD4-PE-Cy7 (clone RM4-5), anti-CD8-APC-Cy7 (clone RAM34) and anti-Flt3-PE (clone A2F10.l), anti-CD3-V450 (clone 17A2), anti-CD4-PECy7 (clone RM4-5), anti-CD8-APC-Cy7 (clone 53-6.7), anti-gamma-APC (clone XMGl.2), and anti-CD44-V500 (clone IM7).
Validation	All antibodies are commercially available and validated by the manufacturer and/or titrated in the lab. Details of validation methods can be found in Supplementary Table 5.

Animals and other research organisms

Policy information about studies involving animals; ARRIVE guidelines recommended for reporting animal research, and Sex and Gender in Research

Laboratory animals	Wild type female C57BL/6 mice were purchased from Charles River Laboratories (Saint Constant, QC, Canada) or the Jackson Laboratory (Bar Harbor, ME). Female Chemokine (C-C motif) receptor 2 (CCR2) (B6.129S4-Ccr2tm1lfc/J), TLR2 (B6.129-Tlr2tm1Kir/J), TLR4 (B6(Cg)-Tlr4tm1.2Karp/J), NOD2 (B6. 129S1-Nod2tm1Flv/J) knock-out and P25 TCR-Tg transgenic mice containing CD4 T cells expressing Ag85B receptor (H2-Kb-Tcra,-Tcrb)P25Ktk/J) on a C57BL/6, background were purchased from the Jackson Laboratory. All mice were 6-8-week of age upon arrival. Mice were housed in a specific pathogen-free level B facility or at the Biosafety Level 3 facility with ad libitum access to food and water, 12hrs light cycle, 50-60% humidity and at 20-250C room temperature at McMaster University, Hamilton. Aged-matched mice and housed in the same room were used in each experiment. Control mice were administered s.c. with PBS used for the preparation of BCG. Animals were assigned experimental groups at random.
Wild animals	no wild animals were used in this study.
Reporting on sex	the findings were applied only to female animals as only the female animals were used.
Field-collected samples	no field collected samples were used in this study.
Ethics oversight	All animal experiments were reviewed NS approved by the Animal Ethics Board (AREB) within McMaster University, Hamilton, Canada on behalf of the laboratory for Dr. Zhou Xing under the animal utilization protocol (AUP) number 210822.

Note that full information on the approval of the study protocol must also be provided in the manuscript.

Flow Cytometry

Plots

Confirm that:

☒ The axis labels state the marker and fluorochrome used (e.g. CD4-FITC).

☒ The axis scales are clearly visible. Include numbers along axes only for bottom left plot of group (a 'group' is an analysis of identical markers).

☒ All plots are contour plots with outliers or pseudocolor plots.

☒ A numerical value for number of cells or percentage (with statistics) is provided.

Methodology

Sample preparation	Mice were euthanized by exsanguination. In some instances, intravascular staining was carried out three minutes before exsanguination by injecting i.v. anti-CD45.2 antibody (clone 104) (BD Pharmingen, San Jose, CA). Cells in bronchoalveolar lavage and lung tissue were isolated. The peritoneal cavity was lavaged. Briefly, 3 ml of total wash solution (PBS containing 2mM EDTA, 1mM HEPES) was injected into the peritoneal cavity and the peritoneum was massaged gently for 30 sec. Lavage fluid was collected with a pipette tip after making small cut in the body wall. Spleen mononuclear cells were obtained. Bone marrow cells were obtained by crushing the spine, femur and tibia bones in a mortar in PBS. Bone marrow cells were then filtered through a 40 µm basket filter (BD Biosciences, San Jose, CA). After lysing red blood cell, isolated cells were resuspended in either complete RPMI 1640 medium (RPMI 1640 supplemented with 10% FBS and 1% L-glutamine, with or without 1% penicillin/streptomycin) for ex vivo culture or in PBS for flow cytometry staining. When the BAL and lung cells were stimulated for intracellular cytokine staining or cultured to measure cytokine/chemokine levels in culture supernatants, cells were resuspended in complete RPMI 1640 medium containing 2% FBS.<br><br>To determine alveolar macrophage activation levels and intracellular cytokine production, 250,000 mononuclear cells from BAL and 2x106 mononuclear cells from lung tissue were plated in flat-bottom 48-well plate and incubated for 3h at 37°C for macrophage adherence and tempering the irrelevant pro-inflammatory activities of freshly isolated alveolar macrophages. At the end of incubation non-adherent cells were washed off and fresh media was added with and without Mtb whole cell lysates (WCL) at a concentration of 1.6?g/ml. To determine levels of trained circulating monocytes and intracellular cytokine production, whole blood was collected into EDTA blood tubes (Sarstedt, Newton, NC) via cardiac puncture and diluted with equal volume of RPMI 1640. Diluted whole blood was aliquoted to 300?l and incubated with or without Mtb whole cell lysates at a concentration of 1.6?g/ml. GolgiPlug (5mg/ml) (BD Biosciences, San Jose, CA) was added to BAL and lung cells and to diluted whole blood cultures 1h after adding the stimulant. Cells were incubated for further 12-14h. To determine activation levels of peritoneal macrophages, 1x106 mononuclear cells were plated in U-bottom 96-well plate and with and without WCL at a concentration of 1.6?g/ml55. GolgiPlug was added 1h after adding the stimulant and the cells were incubated for further 5h. At the end of stimulation, BAL and lung cells were lifted from the wells by incubating in ice cold FACS buffer (0.5% bovine serum albumin) (Sigma Aldrich, Oakville, ON) in the fridge for 15 min. Whole blood mononuclear cells were obtained for immunostaining after incubating with EDTA (Sigma-Aldrich) (30?l of 20 mM EDTA/tube) for 15 min at room temperature and lysing red blood cells using BD Pharm LyseTM (BD Biosciences, San Jose, CA). After staining with Aqua dead cell staining kit (ThermoFisher Scientific Waltham, MA), cells were washed and blocked with anti-CD16/CD32 (clone 2.4G2) and then fixed and permeabilized with BD Cytofix/Cytoperm (BD Biosciences, San Jose, CA) according to manufacturer's instructions.
Instrument	Sample were run on the BD LSR II or BD LSRFortessa flow cytometer at the McMaster Immunology Research Centre.
Software	Flowcytometry data was collected using FACSDiva (Version 8.0)<br>Flowcytometry data was analyzed using FlowJo (Version 10.8.1)
Cell population abundance	Abundance of CD11b+ and CD11c+ cells purified from lung using microbeads (Miltenyi Biotec, Auburn, CA) was 85-90%.<br>Abundance of CD4 T cells from the spleen and lymph nodes of P25-Tg mice were 60-80%.
Gating strategy	Gating strategies used in the study were as per previously published studies (referred in the method section). Briefly, after removing debris, live cells were gated and immune cells were identified using CD45 surface marker. Cell populations were identified using FMO as the guide.

☒ Tick this box to confirm that a figure exemplifying the gating strategy is provided in the Supplementary Information.
